# Diagnostic Value of SFRP1 as a Favorable Predictive and Prognostic Biomarker in Patients with Prostate Cancer

**DOI:** 10.1371/journal.pone.0118276

**Published:** 2015-02-26

**Authors:** Lei Zheng, Dongchen Sun, Wentao Fan, Zhiwei Zhang, Quanlin Li, Tao Jiang

**Affiliations:** 1 Department of Urology, First Affiliated Hospital of Dalian Medical University, Dalian, China; 2 Department of Urology, The fifth People’s Hospital of Dalian, Dalian, China; Northern Institute for Cancer Research, UNITED KINGDOM

## Abstract

Growing genetic and molecular biological evidence suggests that the disruption of balance between Secreted Frizzled-Related Protein-1 (SFRP1) and β-catenin plays an important role in the initiation and development of multiple cancers. The aim of this study was to examine whether the expression of SFRP1 and β-catenin is associated with the clinical-pathologic features of patients with prostate cancer (PCa), and to evaluate their potential roles as predictive and prognostic biomarkers. In this study, a total of 61 patients with PCa and 10 patients with benign prostatic hyperplasia were included, and we showed that the expression of SFRP1 and β-catenin was correlated with the Gleason score, survival rate and response for endocrine therapy of PCa. The survival rates of PCa patients with low SFRP1 expression (P = 0.016) or high β-catenin expression (P = 0.004) were significantly poorer. A negative correlation (r = -0.275, P = 0.032) between SFRP1 and β-catenin was observed by Chi-square test. Multivariate analysis suggested that SFRP1 (hazard ratio, 0.429; 95% confidence intervals, 0.227–0.812; P = 0.009) may serve as an independent predictive and prognostic factor for PCa. We also showed that the protein and mRNA levels of SFRP1 in androgen-dependent PCa cell line LNCaP were significantly higher than those in androgen-independent PCa cell lines DU145 and PC3. However, the protein level of β-catenin in LNCaP cells was significantly lower than that in DU145 and PC3 cells, and no significant difference of β-catenin mRNA level was observed in LNCaP, DU145 and PC3 cells. Bisulfite sequencing PCR assay revealed significantly lower methylation level of *SFRP1* promoter in LNCaP cells than that in DU145 and PC3 cells. Taken together, these findings suggest that SFRP1, which expression inversely correlates with that of β-catenin, is a favorable predictive and prognostic biomarker.

## Introduction

Prostate cancer (PCa) is a common malignant tumor of the urinary system, which is the sixth leading cause of cancer related death in men [1–3], seriously affects the patient’s quality of life. Family history of the disease, ethnicity and advanced age are recognized as the major factors driving PCa, whereas detailed pathogenesis of this disease is still inconclusive [4]. Currently, radical surgical resection, radiation and endocrine therapy are used as treatments of PCa [5–8]. However, this disease is usually fatal for most patients diagnosed in advanced stages. Therefore, it is quite important to identify valuable predictive and prognostic biomarkers for early diagnosis, and to clarify the pathogenesis of PCa.

Wnt proteins are secreted cysteine-rich glycosylated and lipid modified proteins, which play key roles in several cellular processes, including cell differentiation, proliferation, migration, synaptic activity and embryonic development [9–14]. These proteins exert their physiological functions through the Wnt signaling pathway. The Wnt proteins bind to the seven transmembrane (7-Tm) receptors of the frizzled family, and then activate a complex signaling cascade, finally lead to the activation of downstream target genes, such as *c-myc, c-Jun, fibronectin* and *cyclin D1* [15,16]. β-catenin is an important component of the Wnt signaling pathway, whose altered localization has been recognized as a marker of the Wnt signaling pathway activation [16]. In the absence of Wnt ligand, cytoplasmic β-catenin is degraded by the β-catenin destruction complex composed of scaffolding axin protein, casein kinase 1 (CK1), adenomatous polyposis coli (APC) and glycogen synthase kinase 3 (GSK3) through the ubiquitin-proteasome pathway. However, in the presence of Wnt ligand, Wnt ligand binds to its receptor, resulting in the inhibition of β-catenin destruction complex. Subsequently, the unphosphorylated β-catenin molecules accumulate in the cytoplasm, a part of which enter the nucleus and exert their function as a co-activator of LEF/TCF transcription factors, finally lead to the activation of Wnt target genes expression [9]. Currently, increasing evidence suggests that the deregulation of Wnt signaling pathway is closely associated with PCa tumorigenesis in adults [17–20].

Secreted frizzled-related protein-1 (SFRP1), also known as SARP-2, was first isolated from human osteoblast cells, which belongs to the secreted glycoprotein SFRP family. It harbors two distinct structural domains, namely netrin domain and CRD domain. CRD domain is homologous to the frizzled receptors [16,21]. Studies show that SFRP1 is an antagonist of the Wnt signaling pathway, and can bind to Wnt proteins via its CRD domain in a competitive manner with the transmembrane frizzled receptor, leading to the inhibition of the Wnt signaling pathway [22–25]. SFRP1 is required for prostate development, and exerts its role as a stromal-to-epithelial paracrine modulator of epithelial growth, branching morphogenesis, and epithelial gene expression [26]. It is also recognized as a candidate mediator of stromal-to-epithelial signaling in PCa [27]. Inactivation of SFRP1 has been reported due to high methylation of *SFRP1* gene promoter in a variety of malignancies including PCa [28–35]. Moreover, SFRP1 inhibits the transcriptional activity of androgen receptor (AR) and the proliferation of androgen-dependent LNCaP cells [36]. The purpose of this study was to examine the role of SFRP1 and β-catenin expression in human PCa pathogenesis.

In this report, we showed that there was a negative correlation between SFRP1 and β-catenin in human PCa tissues by evaluating clinical-pathologic features. Multivariate analysis revealed that SFRP1 may serve as an independent predictive and prognostic factor for PCa. The protein and mRNA levels of SFRP1 in androgen-dependent PCa cell line LNCaP were significantly higher than those in androgen-independent PCa cell lines DU145 and PC3. However, the protein level of β-catenin in LNCaP cells was significantly lower than that in DU145 and PC3 cells, and no significant difference of β-catenin mRNA level was observed in LNCaP, DU145 and PC3 cells. We also showed that the methylation level of *SFRP1* promoter was significantly lower in LNCaP cells than that in DU145 and PC3 cells. Taken together, our data suggested that SFRP1 is likely to be a favorable predictive and prognostic biomarker.

## Materials and Methods

### Ethics Statement

This study involving human participants was approved by the ethics committee of Dalian Medical University. Written consent was obtained from all the human participants. All research was carried out according to the principles expressed in the Declaration of Helsinki.

### Cell Culture and Experiment Reagents

Human PCa cell lines LNCaP, DU145 and PC3 were obtained from the cell bank of the Shanghai branch of Chinese Academy of Sciences. LNCaP cells were cultured in Roswell Park Memorial Institute (RPMI) 1640 medium supplemented with 10% fetal bovine serum (Gibco, USA), 100 μg/ml penicillin and 100 μg/ml streptomycin. DU145 cells were cultured in Dulbecco’s Modified Eagle’s Medium (DMEM) supplemented with 10% fetal bovine serum (Gibco, USA), 100 μg/ml penicillin and 100 μg/ml streptomycin. PC3 cells were cultured in DMEM/F12 supplemented with 10% fetal bovine serum (Gibco, USA), 100 μg/ml penicillin and 100 μg/ml streptomycin. All cells were incubated at 37°C in a humidified incubator with 5% CO_2_.

Rabbit monoclonal anti-SFRP1 (ab126613) and Mouse monoclonal anti-β-catenin (ab22656) were obtained from Abcam (Cambridge, UK). Mouse monoclonal anti-β-actin (C4) (sc-47778), goat anti-mouse IgG and goat anti-rabbit IgG were obtained from Santa Cruz Biotech (CA, USA). Protease inhibitor mixture was purchased from Roche Applied Science (Basel, Switzerland). 5Aza was obtained from Sigma (Santa Clara, USA). SMARTpool siRNAs (Control and β-catenin) with four individual siRNA targeting a single gene were obtained from Thermo (USA).

### PCa tissue samples for immunohistochemical assay

A total of 71 samples were obtained from First Affiliated Hospital of Dalian Medical University, Liaoning, China from 2002 to 2008, including 61 samples from patients that were pathologically or cytologically verified adenocarcinoma of the prostate and 10 benign prostatic hyperplasia samples. All PCa specimens were from patients with ages ranging from 44 to 84 (average age of 72 years). The tumor grades were recorded as Gleason score>7 (34 specimens), and Gleason score≤7 (27 specimens). According to tumor node metastasis (TNM) system developed by the American Joint Committee on Cancer (AJCC), all patients participated in this research were in T_3_/T_4_/N_x_-1 M_0_ stage. Benign prostatic hyperplasia samples were obtained from patients for treatment of non-tumor diseases, with ages ranging from 48 to 81 (average age of 71.8 years). All patients were followed up to December 2013. The survival time ranged from 3 to 60 months, with a median time of 38 months. During this period, 38 patients died due to cancer recurrence.

Evaluation criteria of response for endocrine therapy: (1) Effective endocrine therapy. After endocrine treatment (medicines or surgical castration), PSA level returns to normal and <2 ng/ml within three years. No new metastatic lesions appear in bone through CT, MRI or ECT scanning; (2) Ineffective endocrine therapy. After endocrine treatment, PSA level does not reduce, or transiently decreases and then increases. Disease aggravates, and new metastatic lesions appear in bone in two years.

### Immunohistochemical Assay

All samples were analyzed by immunohistochemistry. The specimens excised by operation were fixed in 10% buffered formalin for 24 h, and then the fixed tissues were embedded in paraffin. Four micrometers sections of the successive paraffin-embedded samples were cut for immunohistochemical analysis with the aid of a Histostain-Plus kit (Invitrogen, Camarilo, CA). All sections were deparaffinized with xylene and rehydrated with graded ethanol solution. H_2_O_2_ (3%) was used to quench the endogenous peroxidases of the specimens, and then the sections were incubated in blocking serum for 10 min at 37°C. The sections were then stained with citrate buffer (pH 6.0) containing either anti-SFRP1 or anti-β-catenin at a dilution of 1:100 overnight at 4°C. The sections were then incubated with biotinylated goat anti-rabbit immunoglobulin at a dilution of 1:200 for 10 min at room temperature, and then incubated with Avidin-Biotin Peroxidase Complex for 10 min at room temperature. Finally, the sections were incubated with DAB (diaminobenzidine) used as chromogen.

SFRP1-positive staining cases exhibited clear brown yellow granules in cytoplasm. β-catenin-positive staining cases exhibited clear brown yellow granules in cytomembrane and nucleus. Scoring of the staining was made according to the following criteria: (1) Scoring based on staining intensity. 0, no cell staining; 1, cells with pale yellow; 2, cells with pale brown; 3, cells with brown. (2) Scoring based on percentage of positive cells. Samples were observed under high power magnification (400×). 10 random fields were selected, and 100 cells per field were counted. 0, <25% cell staining; 1, 25%∼50% cell staining; 2, 51%∼75% cell staining; 3, >75% cell staining. Taken together, (1) + (2) >3 was recognized as positive samples, whereas (1) + (2) ≤3 was recognized as negative samples.

### Western Blot Assay

LNCaP, DU145 and PC3 cells treated with or without 5Aza (DNA methylation inhibitor) were harvested and lysed in a cold buffer (50 mM Tris–HCl, pH 8.0, 150 mM NaCl, 1% Nonidet P-40, 0.5% sodium deoxycholate, and protease inhibitor mixture). After centrifuging at 12000 rpm for 10 min at 4°C, the samples of supernatant were subjected to SDS-PAGE. Then, the protein bands in gel were transferred to poly vinylidene fluoride (PVDF) membrane (Millipore), and probed with indicated primary antibody overnight at 4°C. Next, the PVDF membrane was incubated with the appropriate secondary antibody for 4 h at room temperature. The results were analyzed by chemiluminescence detection. Data were quantified by scanning the appropriate bands of interest and plotted as relative density of gray scale. The experiment was replicated three times.

### Quantitative RT-PCR Assay

Total RNA from each cell line (LNCaP, DU145 and PC3) was extracted using RNAiso Plus reagent (Takara, Dalian, China) according to the manufacturer’s instruction. Reverse transcription was performed with the aid of a reverse transcription kit (Takara, Dalian, China). Real-time PCR was performed with the LightCycler Real-Time PCR System (Roche Diagnostics, Basel, Switzerland). The following primers designed by Primer Premier 5.0 were used: 5’-CCCGAGATGCTTAAGTGTGACAA-3’ (sense) and 5’-ACTCGCTGGCACAGAGATGTTC-3’ (antisense) for *SFRP1*; 5’-AGAAAAGCGGCTGTTAG-3’ (sense) and 5’-ATACAGGACTTGGGAGGT-3’ (antisense) for *β-catenin*; 5’-CAGGTCATCACTATCGGCAA-3’ (sense) and 5’-CAAAGAAAGGGTGTAAAACGC-3’ (antisense) for *β-actin*. The samples containing cDNA, the appropriate pair of primers and the maxima SYBR Green qPCR Master Mix (Thermo) were subjected to the following reaction: initial denaturation step of 95°C for 10 min; and 40 cycles of 95°C for 30 s, 55°C for 15 s, and 72°C for 20 s. The 2^-△△CT^ method was used for data analysis. The mRNA levels of *SFRP1* and *β-catenin* were normalized to *β-actin*, which is served as the endogenous control.

### Bisulfite sequencing PCR Assay (BSP)

DNA from each cell line (LNCaP, DU145 and PC3) was extracted using a DNA purification kit (Tiangen, Beijing, China) according to the manufacturer’s recommendations. The isolated DNA was treated with sodium bisulfite using the CpGenome Fast DNA Modification Kit (Millipore) according to the manufacturer’s recommendations. The DNA treated with sodium bisulfite was amplified by a GeneAmp 9600 PCR System (Applied Biosystems, Foster City, CA, USA). The following primers designed by Primer Premier 5.0 were used: 5’-GGTTAAGGTAGGAGTATTATTTGAGGT-3’ (sense) and 5’-AACCTAAATCATACTTACAAACCCAT-3’ (antisense) for CpG island 1 of *SFRP1* promoter; 5’-TTGTTTTTTAAGGGGTGTTGAGT-3’ (sense) and 5’-TACCACAAACTTCCAAAAACCTC-3’ (antisense) for CpG island 2 of *SFRP1* promoter. The following reaction conditions were performed: 10 cycles of 95°C for 5 min, 95°C for 30 s, 60°C-50°C for 45 s (beginning at 60°C for the first cycle but with the temperature decreasing by 1°C for each subsequent cycle), and 72°C for 45 s; 33 cycles of 95°C for 30 s, 50°C for 45 s, and 72°C for 45 s; and 60°C for 30 min. The PCR products were purified using a DNA purification kit (Tiangen, Beijing, China), and were cloned in pMD-19T vector (Takara, 6013). After transformation, 10 positive clones of each BSP sample were chosen for sequencing using a 3730xl DNA analyzer (Applied Biosystems).

### Statistical Analysis

A Chi-square (*χ* ^2^) test was used to clarify the correlation between SFRP1 and β-catenin expression and clinical-pathologic features in PCa tissues from 71 patients. Patient survival rates were analysed by the Kaplan-Meier method. Spearman rank correlation was carried out to evaluate the relationship between SFRP1 and β-catenin expression. Data were expressed as means ±SDs. Differences between mean values were analysed by ANOVA followed by multiple comparison Student-Neuman-Keuls tests and bi-variant relationship analyses. Statistical significance was considered at *P*<0.05 level.

## Results

### The expression of SFRP1 and β-catenin is associated with clinical-pathologic features of human PCa

Aberrant expression of SFRP1 and β-catenin plays important roles in the development of a range of cancers, such as biliary tract cancer, mucoepidermoid carcinoma, adrenocortical tumors and cervical cancer [16,37–39]. However, the role of SFRP1 and β-catenin in PCa has not been elucidated. In order to investigate their roles in PCa, we examined whether the expression of SFRP1 and β-catenin correlated with clinical-pathologic features among the 61 PCa samples by *χ* ^2^ test. Statistically significant differences were observed in the Gleason score, survival rate and response for endocrine therapy for the two proteins, whereas no statistically significant difference was observed in the age, metastasis before surgery and PSA level (Table 1). Taken together, these results suggested that the expression SFRP1 and β-catenin correlated with the Gleason score, survival rate and response for endocrine therapy of PCa patients, whereas didn’t correlate with the age, metastasis before surgery and PSA level.

**Table 1:** Correlations between the SFRP1/β-catenin expression and clinical-pathologic features in human PCa.

Clinical-pathologic features	n	SFRP1		Positive rate (%)	*P*	β-catenin		Positive rate (%)	*P*
		-(n = 25)	+ (n = 36)			-(n = 32)	+(n = 29)		
**Age (years)**									
≤72	23	12	11	47.8%	0.170	9	14	60.9%	0.108
>72	38	13	25	65.8%		23	15	39.5%	
**Gleason score**									
≤7	27	7	20	74.1%	**0.035**	20	7	25.9%	**0.003**
>7	34	18	16	47.1%		12	22	64.7%	
**Metastasis before surgery**									
+	35	16	19	54.3%	0.387	15	20	57.1%	0.084
-	26	9	17	65.4%		17	9	34.6%	
**Survival rate**									
>5 years	22	5	17	77.3%	**0.031**	16	6	27.3%	**0.018**
≤5 years	39	20	19	48.7%		16	23	59.0%	
**PSA**									
≤20 ng/ml	12	5	7	58.3%	0.958	4	8	66.7%	0.142
>20 ng/ml	49	20	29	59.2%		28	21	42.9%	
**Response for endocrine therapy**									
+	23	6	17	73.9%	**0.027**	16	7	63.6%	**0.027**
-	22	13	9	40.9%		8	14	30.4%	

### The effect of SFRP1 expression on survival rate in PCa patients

**Fig 1:**
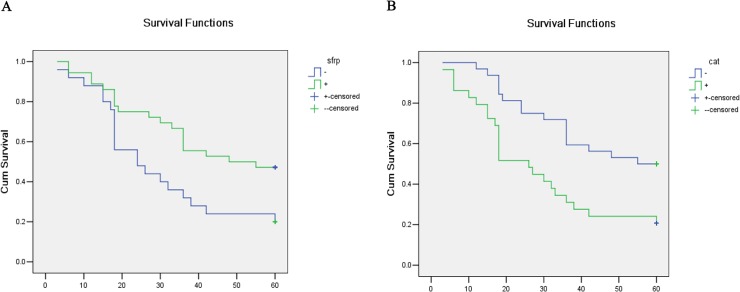
Kaplan-Meier curves of the relationship of SFRP1 and β-catenin expression with overall survival in PCa patients. A, The relationship of SFRP1 expression with overall survival in patients with PCa (P = 0.016). B, The relationship of β-catenin expression with overall survival in patients with PCa (P = 0.004).

To further determine the relationship between SFRP1/β-catenin expression and overall survival in PCa, survival curves were drawn by the Kaplan-Meier method. Results revealed that SFRP1-negative patients showed a significantly poorer overall survival rate than SFRP1-positive patients (P = 0.016, Fig. 1A). In contrast, β-catenin-positive patients showed a significantly poorer overall survival rate than β-catenin-negative patients (P = 0.004, Fig. 1B). These results suggested that negative SFRP1 expression and positive β-catenin expression seriously affected the overall survival rate of patients with PCa.

To evaluate the contribution of clinical-pathologic features as potential predictive and prognostic biomarkers, we analyzed 6 clinical-pathologic variables in the 61 PCa patients by multivariate analysis. As shown in Table 2, Only SFRP1 expression was statistically significant factor (P = 0.009), and could be identified as an independent predictive and prognostic indicator, indicating that SFRP1 expression may serve as the best predictive and prognostic biomarker of survival rate in PCa among these variables.

**Table 2:** Multivariate Cox regression analysis of prognostic biomarkers in 61 PCa patients.

Factors	Category	X^2^	P	HR*	95% CI*
Age (years)	≤72	0.353	0.552	0.988	0.949–1.028
	>72				
Gleason score	≤7	0.227	0,634	1.220	0.538–2.763
	>7				
Metastasis before surgery	+	3.659	0.056	1.966	0.983–3.928
	-				
PSA	≤20ng/ml	0.302	0.583	0.785	0.331–1.863
	>20ng/m				
SFRP1 expression	positive	6.755	**0.009**	0.429	0.227–0.812
	negative				
β-catenin expression	positive	2.216	0.137	0.593	0.298–1.180
	negative				

*HR hazard ratio, CI confidence interval.

### The expression of SFRP1 and β-catenin is negatively correlated in PCa

**Fig 2:**
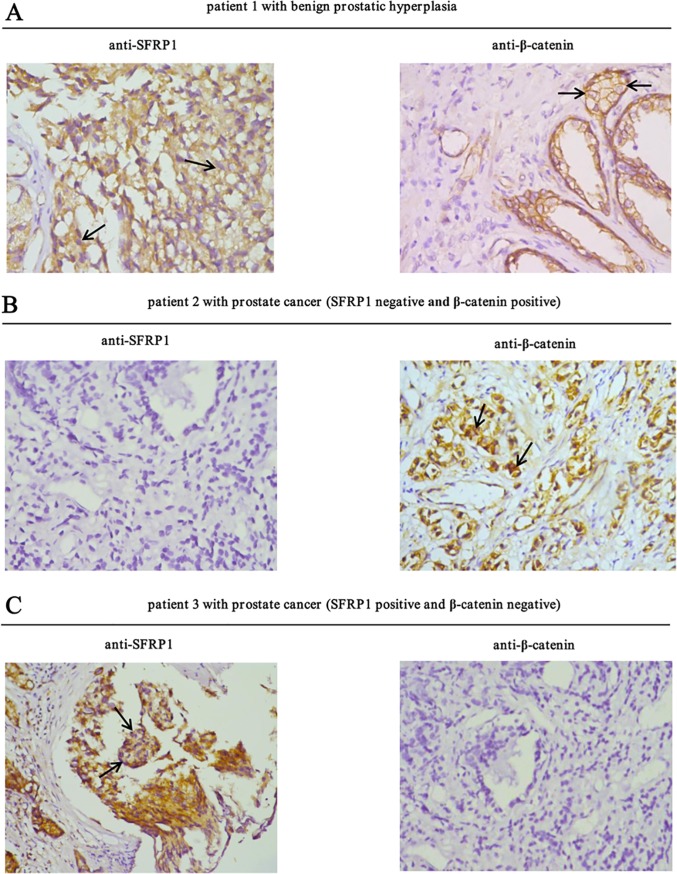
Correlation between SFRP1 and β-catenin expression in human benign prostatic hyperplasia and PCa samples. A, Representative results showing the immunohistochemical staining of SFRP1 and β-catenin in a section of human benign prostatic hyperplasia tissues. In the left panel, arrows show that the localization of SFRP1 uniformly distributed in the cytoplasm. In the right panel, arrows show that the localization of β-catenin was predominately cell membrane. B, The immunohistochemical staining of SFRP1 and β-catenin in a section of human prostate cancer tissues (SFRP1 negative and β-catenin positive). In the right panel, arrows show that β-catenin mainly localized in both cytoplasm and nucleus. C, The immunohistochemical staining of SFRP1 and β-catenin in a section of human prostate cancer tissues (SFRP1 positive and β-catenin negative). In the left panel, arrows show that the localization of SFRP1 was predominately in the cytoplasm.

To determine the correlation between SFRP1/β-catenin expression and occurrence of PCa, the protein expression of SFRP1 and β-catenin in benign prostatic hyperplasia and prostate tumor samples were compared by immunohistochemistry (Fig. 2). A total of 71 tissue samples (10 benign prostatic hyperpslasia and 61 prostate tumors) were analyzed. According to the rating criteria, 8 (80%) of the 10 benign prostatic hyperplasia samples were classified as positive for SFRP1 expression and 2 (20%) were classified as negative, whereas in tumor samples, SFRP1 expression significantly decreased with 36 (59%) positive and 25 (41%) negative. In contrast, in benign prostatic hyperplasia samples, 9 (90%) were classified as negative for β-catenin expression and 1 (10%) was classified as positive expression, whereas β-catenin expression significantly increased with 29 (47.5%) positive and 32 (52.5%) negative in tumor samples (Table 3). To prove the specificity of β-catenin antibody, siRNA knockdown experiment was conducted using PC3 cells. The endogenous β-catenin expression was decreased by 86% in PC3 cells transfected with siβ-catenin pool compared with PC3 cells transfected with siControl (S1 Fig). These results indicated that SFRP1 expression was decreased, whereas β-catenin expression was increased in PCa.

**Table 3:** Immunohistochemical staining of SFRP1 and β-catenin proteins in human PCa versus benign prostatic hyperplasia.

Prostate status	n	SFRP1		β-catenin	
		Negative	Positive	Negative	Positive
Cancerous	61	25 (41%)	36 (59%)	32 (52.5%)	29 (47.5%)
Non-cancerous	10	2 (20%)	8 (80%)	9 (90%)	1 (10%)

To examine the relationship between SFRP1 and β-catenin in PCa tissues, the protein levels of SFRP1 and β-catenin in prostate tumor samples were analysed by chi-square tests. According to our rating criteria, 36 samples were identified as SFRP1-positive expression, and 25 were identified as SFRP1-negative expression in the 61 PCa samples. 23 (63.9%) of the SFRP1-positive samples showed negative β-catenin expression, whereas only 9 samples (36%) showed negative β-catenin expression in SFRP1-negative samples (Table 4). These results showed that there was a negative correlation between SFRP1 and β-catenin in PCa (r = -0.275, P = 0.032).

**Table 4:** Inverse correlation between SFRP1 expression and β-catenin expression in human PCa*.

Protein status	β-catenin negative	β-catenin positive	Total
**SFRP1 negative**	9 (14.8%)	16 (26.2%)	25
**SFRP1 positive**	23 (37.7%)	13 (21.3%)	36
**Total**	32	29	61

*r = -0.275, *P* = 0.032.

**Fig 3:**
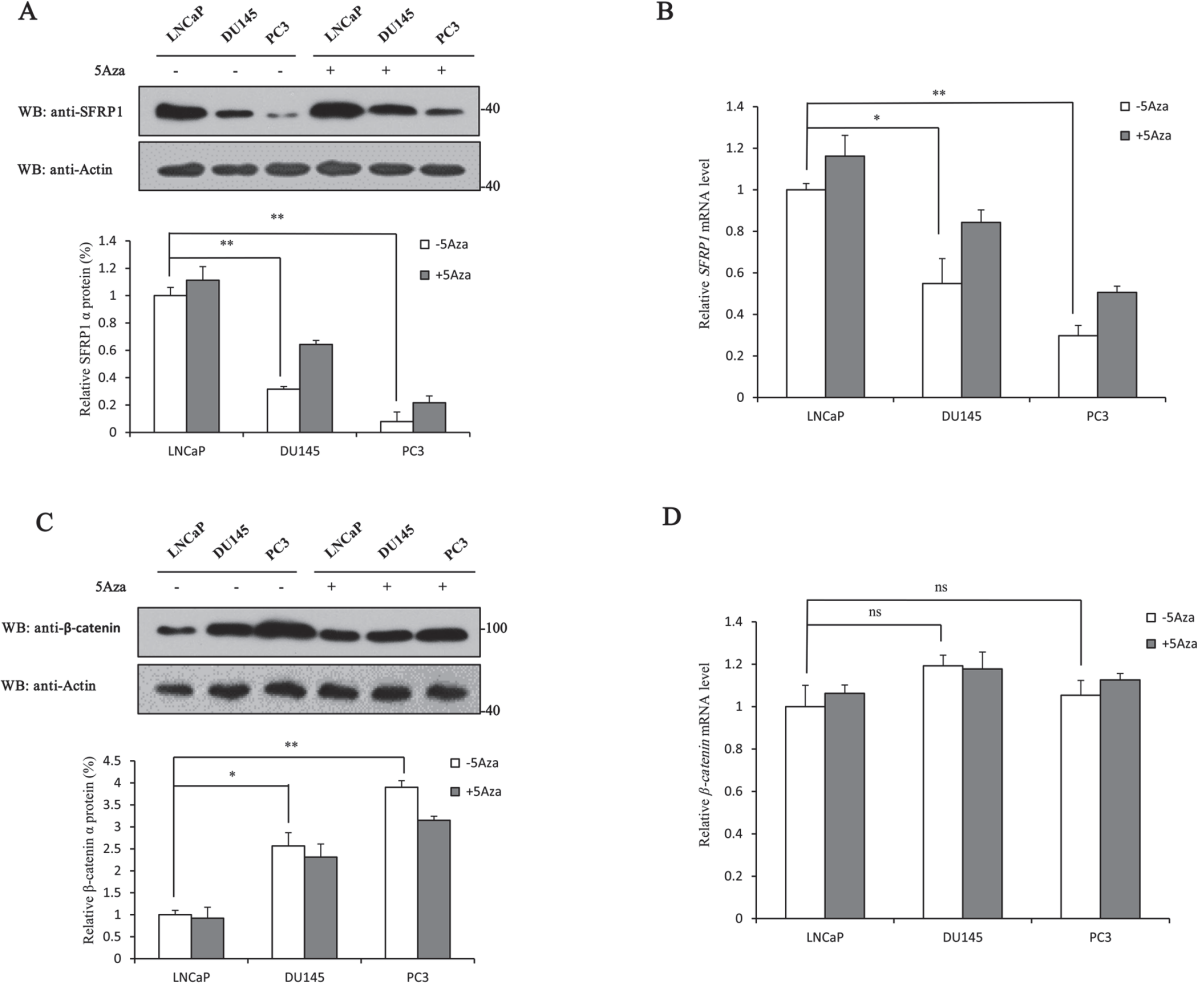
5Aza-induced changes in protein and mRNA levels of SFRP1 and β-catenin in PCa cell lines. A, LNCaP, DU145 and PC3 cells treated with or without 5Aza were collected and then subjected to Western blot analysis with anti-SFRP1 antibody. B, LNCaP, DU145 and PC3 cells treated with or without 5Aza were collected and then subjected to quantitative RT-PCR assay. The *SFRP1* mRNA level of LNCaP cells was set to 1. C, LNCaP, DU145 and PC3 cells treated with or without 5Aza were collected and then subjected to Western blot analysis with anti-β-catenin antibody. D, LNCaP, DU145 and PC3 cells treated with or without 5Aza were collected and then subjected to quantitative RT-PCR assay. The *β-catenin* mRNA level of LNCaP cells was set to 1. All experiments were repeated at least three times. Data shown in the graphs are the means ±SDs of three experiments. *, *P*<0.05. ns, not significant. The full-length blot of Fig. 3A is presented in S2 Fig.

To further determine the change of SFRP1 and β-catenin expression in different PCa cell lines, the protein and mRNA levels of SFRP1 and β-catenin in LNCaP, DU145 and PC3 cells treated with or without 5Aza were examined. Among them, LNCaP is an androgen-dependent human prostate cancer cell line, while DU145 and PC3 are androgen-independent human prostate cancer cell lines, which are more highly malignant than LNCaP cells. With the malignant degree increased gradually, the protein and mRNA levels of SFRP1 in the PCa cells treated without 5Aza significantly decreased, whereas both protein and mRNA levels of SFRP1 were up-regulated in the PCa cells treated with 5Aza (Fig. 3A&3B). However, the protein level of β-catenin significantly increased in the PCa cells treated without 5Aza, and was down-regulated in the PCa cells treated with 5Aza (Fig. 3C). No significant difference of β-catenin mRNA level was observed in LNCaP, DU145 and PC3 cells (Fig. 3D). Taken together, these results confirmed that there was an inverse correlation between SFRP1 and β-catenin expression in PCa cell lines, which is consistent with the data obtained from tumor tissue samples.

### The changes of *SFRP-1* gene methylation in PCa cell lines

**Fig 4:**
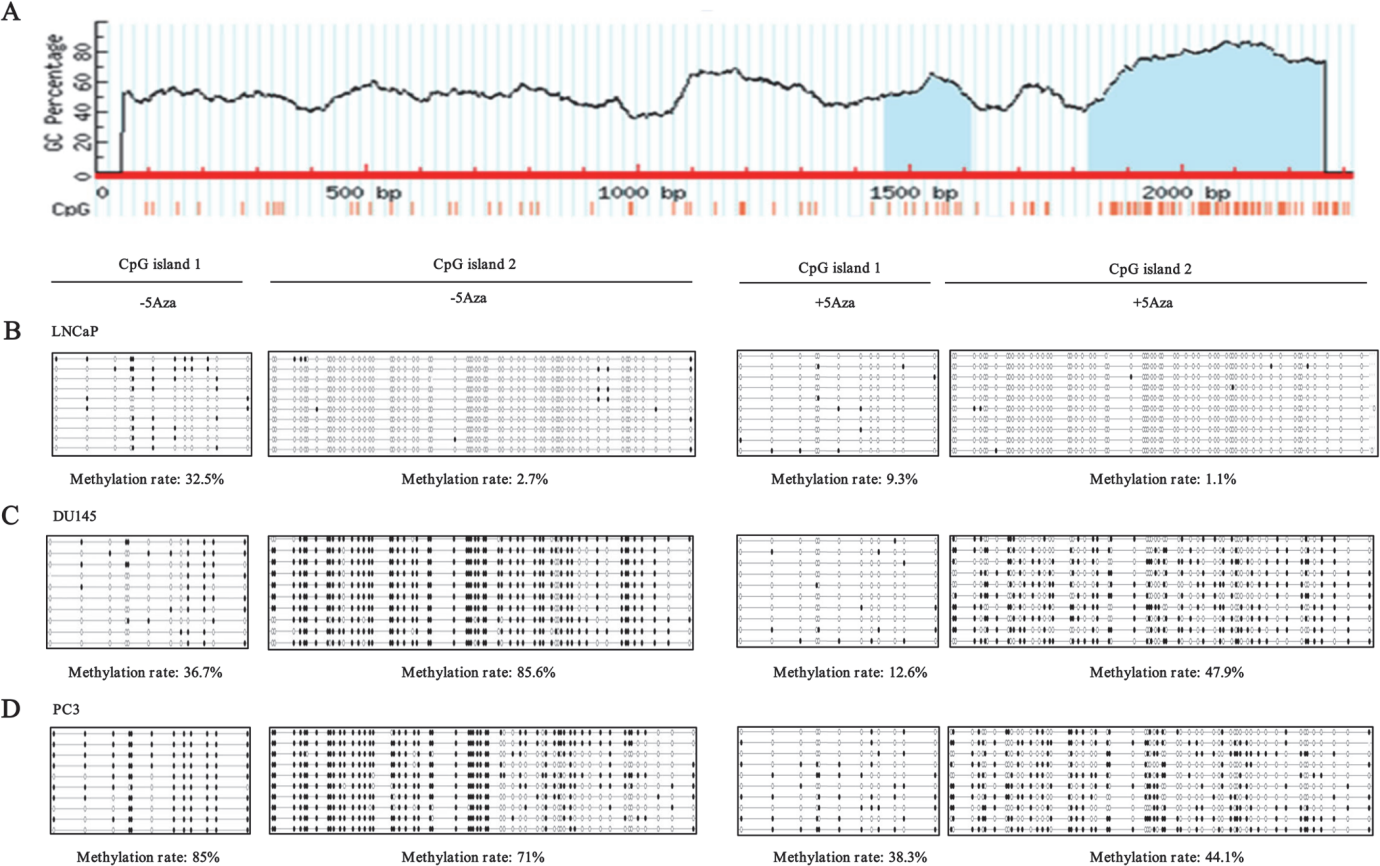
Changes of *SFRP-1* promoter methylation in LNCaP, DU145 and PC3 cells. A, Schematic diagram of the SFRP-1 promoter region (blue-colored regions: CpG islands). CpG island 1 was from 1454 bp to 1613 bp. CpG island 2 was from 1831 bp to 2257 bp. B-D, Methylation of CpG island 1 (left panel) and CpG island 2 (right panel) in LNCaP, DU145 and PC3 cells, respectively treated with or without 5Aza. Each circle represents a methylated (black) or unmethylated (white) CpG dinucleotide. Every row represents a different clone.

The methylation of *SFRP1* gene promoter has been reported to lead to down-regulation of SFRP1 protein and mRNA levels, and to play important roles in the initiation and development of tumors. Given that the protein and mRNA levels of SFRP1 significantly decreased with increasing degree of malignancy, and 5Aza, a DNA methylation inhibitor, up-regulated both protein and mRNA levels of SFRP1 in PCa cell lines, we speculated that SFRP1 silencing was likely to be induced by *SFRP1* gene methylation. To investigate this possibility, the methylation of *SFRP1* gene promoter in LNCaP, DU145 and PC3 cells treated with or without 5Aza was examined by BSP assay. Methylation usually occurs in CpG-rich promoter regions, named CpG island with island size>100, GC%>50, Obs/Exp>0.6. Bioinformatics analysis showed that 2 CpG islands might be methylated in *SFRP1* gene promoter (Fig. 4A, blue-colored regions). BSP assay showed that both CpG islands in the *SFRP-1* promoter were methylated in LNCaP, DU145 and PC3 cells. The methylation rates of CpG islands 1 and 2 in LNCaP cells treated without 5Aza were 32.5% and 2.7% respectively, whereas both of the methylation rates dropped to 9.3% and 1.1% after 5Aza treatment (Fig. 4B). In DU145 cells, the methylation rates of CpG islands 1 and 2 declined from 36.7% and 85.6% to 12.6% and 47.9%, respectively after 5Aza treatment (Fig. 4C). In PC3 cells, both of the methylation rates declined from 85% and 71% to 38.3% and 44.1% after 5Aza treatment (Fig. 4D). These results corresponded with the data obtained from Western blot analysis (Fig. 3A&3B), suggesting that the down-regulation of protein and mRNA levels of SFRP1 was induced by *SFRP1* gene methylation with increasing degree of malignancy of PCa cell lines.

## Discussion

PCa is the most commonly diagnosed non-skin cancer in men with increased incidence rate every year [40–42]. The pathological features of PCa are immensely complex [43]. Tumors in many patients with PCa are in an indolent form, which have no effect on the survival of patients and are frequently kept under surveillance rather than immediate treatment, whereas a small proportion of tumors belong to rapidly progressing malignant tumors, which seriously threaten the life of patients [44,45]. In addition, owing to the physiological and anatomical features of PCa, it is difficult to observe obvious early symptoms of patients, leading to a large number of patients diagnosed at an advanced stage [46]. Therefore, it is important to improve early diagnosis for PCa through identifying predictive and prognostic biomarkers. In this study, we demonstrated that the Wnt antagonist SFRP1 was a favorable predictive and prognostic biomarker and its expression was inversely correlated with β-catenin expression.

Wnt signaling pathway plays an important role in several cellular processes, such as cell differentiation, proliferation, and migration, and its deregulation correlates with several diseases including cancer [47]. The Wnt/β-catenin signaling pathway is abnormally activated in a wide range of human malignancies [48–54]. This aberrant activation contributes to tumorigenesis by up-regulating the expression of various downstream target genes, such as *MDR-1, Livin, cyclin D1* and *c-Myc* [15,55]. β-catenin is a core component of the Wnt signaling pathway, and its altered localization is recognized as a marker of pathway activation. The data from immunohistochemical staining showed that β-catenin localization in human benign prostatic hyperplasia samples was predominantly localized to the cell membrane with low protein expression levels (Fig. 2A right panel); however, in human PCa samples, β-catenin mainly localized in the cytoplasm and nucleus with higher protein expression amount compared with that in benign prostatic hyperplasia samples (Fig. 2B right panel). It has been reported that β-catenin interacts with AR and enhances the androgen-stimulated transcriptional activity of AR, suggesting that the role of β-catenin in prostate carcinogenesis may not be limited to transcriptional activation of TCF/LEF [56]. In this study, we showed that β-catenin expression was positive in 47.5% of PCa samples, whereas only 10% (1/10) positive expression was observed in benign prostatic hyperplasia samples (Table 2). The positive expression of β-catenin in PCa was also significantly correlated with Gleason score, survival rate and response for endocrine therapy (Table 1). We demonstrated that PCa patients with positive β-catenin expression had a significantly poorer overall survival rate (Fig. 1B). Moreover, the expression of β-catenin significantly up-regulated with increasing degree of malignancy (Fig. 3C). These results suggest that the expression of β-catenin is closely related to poorer prognosis, and that aberrant activation of the Wnt signaling pathway contributes to the development of PCa.

SFRP1 exerts its function in the Wnt signaling pathway as a well-known antagonist of the frizzled receptor, and has been suggested to be a tumor suppressor in several human cancers [57,58]. The data from immunohistochemical staining showed that the localization of SFRP1 uniformly distributed in the cytoplasm (Fig. 2A left panel), consistent with its expression in several other tissues [34,59]. However, SFRP1 localization is predominately cytoplasmic perinuclear in Biliary tract and bladder cancers [16,60]. The difference in the distribution of SFRP1 may be due to tissue-specific expression. Silencing of SFRP1 was observed in a wide range of malignancies [26–36], indicating that loss of SFRP1 is likely to be a common mechanism to activate the Wnt signaling pathway in human cancers. We observed that the SFRP1 expression was reduced in PCa samples compared with that in benign prostatic hyperplasia samples from 80% to 59% (Table 2) and its expression was inversely correlated with β-catenin expression (Table 3), consistent with their expression patterns in other tumor samples [16,37]. In addition to inhibiting the Wnt pathway, SFRP1 was reported to inhibit the transcriptional activity of AR and the proliferation of androgen-dependent LNCaP cells [36]. Therefore, down-regulation of SFRP1 expression may also reduce its ability to inhibit AR transcriptional activity, leading to the development of PCa. We also showed here that the expression of SFRP1 was significantly correlated with Gleason score, survival rate and response to endocrine therapy (Table 1). PCa patients with negative SFRP1 expression had a significantly poorer overall survival rate (Fig. 2A). Moreover, multivariate analysis suggested that SFRP1 (hazard ratio, 0.429; 95% confidence intervals, 0.227–0.812; P = 0.009) may serve as an independent predictive and prognostic factor for PCa. In addition, we showed here that the protein and mRNA levels of SFRP1 are significantly down-regulated with increasing degree of PCa cell line malignancy (Fig. 3A&3B). This is consistent with the data from another study [61]. Taken together, these results imply that SFRP1 possesses the potential to be a favorable predictive and prognostic biomarker for PCa treatment.

The detailed molecular mechanism of SFRP1 silencing has not been clarified. However, growing evidence shows that SFRP1 expression is tightly regulated by promoter methylation. Related studies have been described in a wide range of malignant tumors, including PCa [28–35]. In this study, we observed that the SFRP1 promoter was methylated in PCa cell lines LNCaP, DU145 and PC3, and the methylation level was up-regulated with increasing degree of malignancy. After 5Aza treatment, the methylation level in the three PCa cell lines was down-regulated. These results corresponded with the data from Western blot analysis (Fig. 3A&3B), suggesting that the down-regulation of SFRP1 protein and mRNA levels was induced by SFRP1 promoter methylation.

In conclusion, we showed that the expression of SFRP1 and β-catenin was associated with the Gleason score, survival rate and response for endocrine therapy of PCa. In addition, a negative correlation between SFRP1 and β-catenin was observed, and that negative SFRP1 expression and positive β-catenin expression were linked to the overall survival rate of PCa patients. Moreover, with increasing degree of malignancy, the protein and mRNA levels of SFRP1 significantly decreased, whereas the protein level of β-catenin significantly increased, and no significant change of its mRNA level was observed in PCa cell lines. Furthermore, methylation of *SFRP1* promoter significantly increased, with increasing degree of malignancy of cell lines. These findings suggested that SFRP1 has the potential to be a favorable predictive and prognostic biomarker for PCa. SFRP1 as a secreted protein exerts its function outside of the cells. Therefore, SFRP1 may also play roles in tumorigenesis of PCa through other signalling pathways in addition to the Wnt signalling pathway. More research is required to address the mechanism of SFRP1 action in the development of PCa.

## Supporting Information

S1 Fig The effect of siβ-catenin on endogenous β-catenin expression in PC3 cells. PC3 cells transfected with siβ-catenin pool (with four individual siRNAs targeting *β-catenin* gene) or control siRNA (siControl) were collected and then subjected to Western blot analysis with anti-β-catenin antibody and anti-β-actin antibody.
(TIF)

S2 Fig The full-length blot of Fig. 3A. LNCaP, DU145 and PC3 cells treated with or without 5Aza were collected and then subjected to Western blot analysis with anti-SFRP1 antibody and anti-β-actin antibody.
(TIF)
