# Supplementary figures and images for: Diagnostic Value of SFRP1 as a Favorable Predictive and Prognostic Biomarker in Patients with Prostate Cancer
Source: PLoS One. 2015 Feb 26;10(2):e0118276. doi: 10.1371/journal.pone.0118276 (PMC4342152; doi:10.1371/journal.pone.0118276)

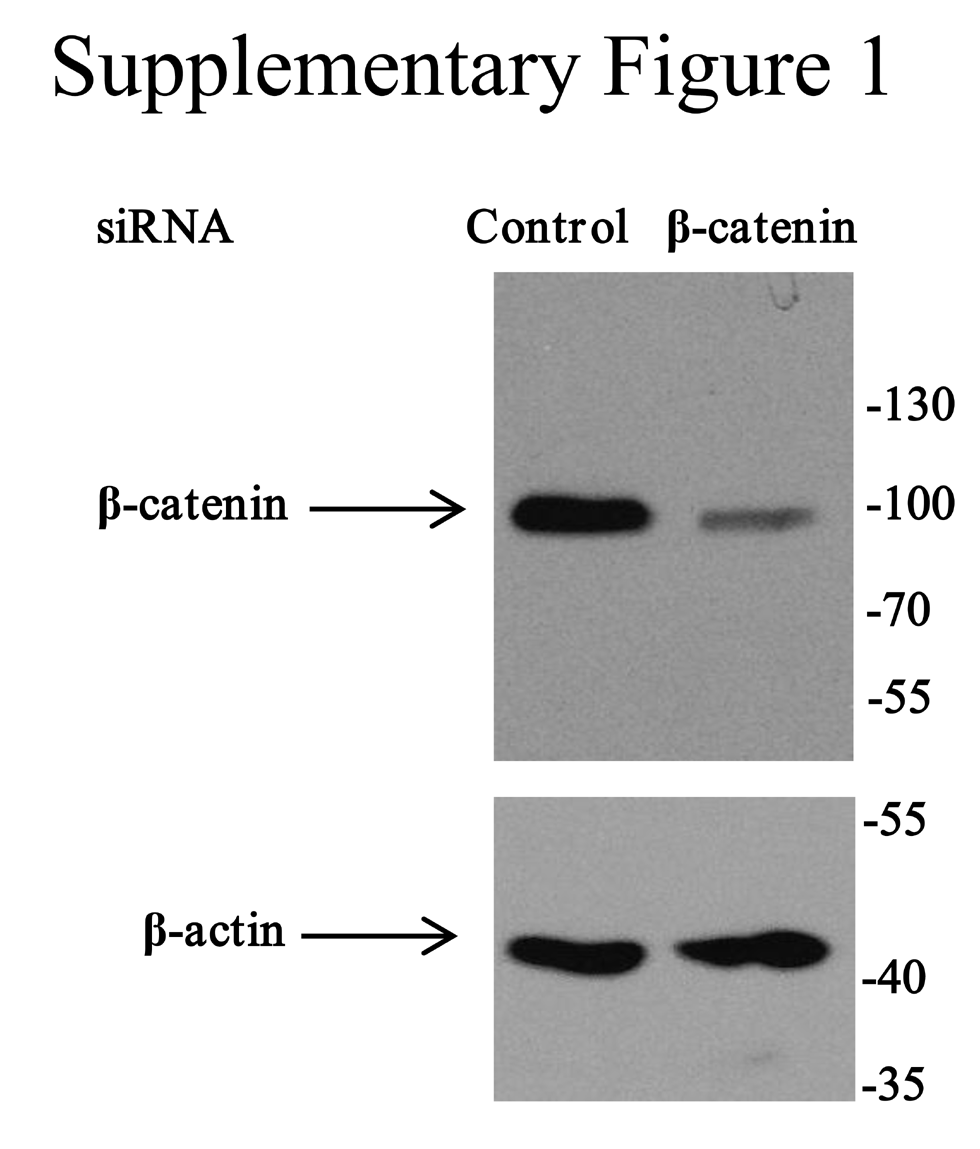

Supplement: S1 Fig — PC3 cells transfected with siβ-catenin pool (with four individual siRNAs targeting β-catenin gene) or control siRNA (siControl) were collected and then subjected to Western blot analysis with anti-β-catenin antibody and anti-β-actin antibody. (TIF) [file pone.0118276.s001.TIF]

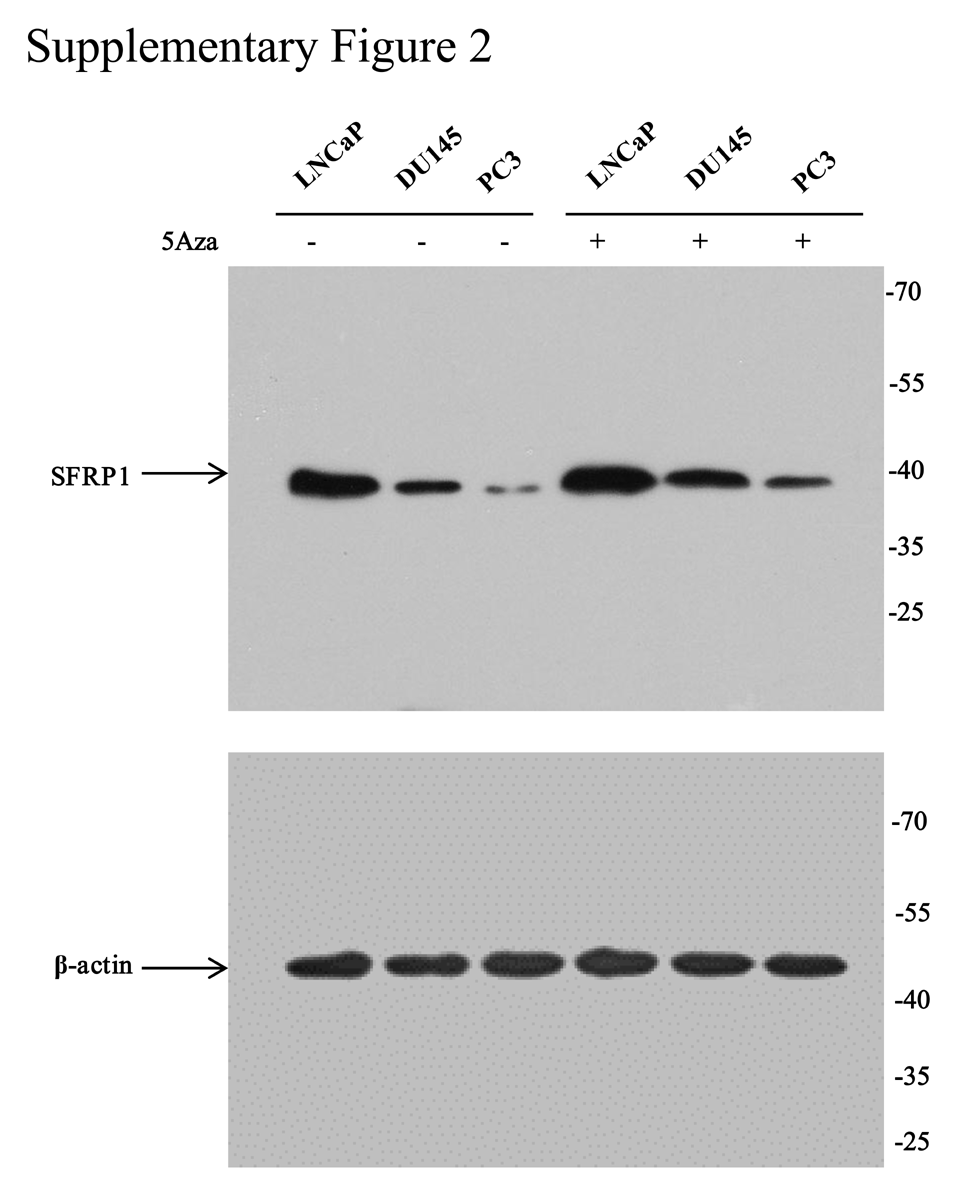

Supplement: S2 Fig — LNCaP, DU145 and PC3 cells treated with or without 5Aza were collected and then subjected to Western blot analysis with anti-SFRP1 antibody and anti-β-actin antibody. (TIF) [file pone.0118276.s002.TIF]
